# Global surveillance of circulating microRNA for diagnostic and prognostic assessment of acute myocardial infarction based on the plasma small RNA sequencing

**DOI:** 10.1186/s40364-024-00690-x

**Published:** 2024-11-19

**Authors:** Xiaomin Wang, Yaojun Lu, Ruiping Zhao, Bing Zhu, Jian Liu, Qiang Yue, Rina Wu, Shuwen Han, Yuanyuan Gao, Juan Chen, Jie Gong, Danna He, Teng Xu, Jianchao Ying

**Affiliations:** 1 Department of Cardiology/Chest Pain Center, Baotou Central Hospital, Baotou, China; 2 Institute of Translational Medicine, Baotou Central Hospital, Baotou, China; 3 Dian Diagnostics Group Co., Ltd, Hangzhou, China; 4 Wenzhou Key Laboratory of Emergency, Critical Care, and Disaster Medicine/Central Laboratory, The First Affiliated Hospital of Wenzhou Medical University, Wenzhou, China

**Keywords:** Acute myocardial infarction, Circulating microRNA, Diagnostic biomarker

## Abstract

**Background:**

Circulating microRNAs (miRNAs) are recently a rapidly increasing of interest as non-invasive biomarkers for diagnosis and prognosis of acute myocardial infarction (AMI). Previous studies revealed that several miRNAs exhibited the capacity for diagnosis and prognosis of AMI, the reasons why these circulating miRNAs are concerned as targets for investigation are quite cryptogenic, presumably due to the lack of clues provided by global surveillance at the transcriptome level, and the current data for some miRNAs are controversial and inconsistent among independent studies.

**Methods:**

To comprehensively profiling the potential miRNAs for diagnosis and prognosis of AMI, we reported transcriptomes of circulating miRNAs in the plasma of 27 healthy controls, 64 AMI patients (37 STEMI and 27 NSTEMI) and 20 AMI patients who were subjected to reperfusion therapy. Meanwhile, the cTnI of AMI patients was parallel determined. Differentially-circulated miRNAs were analyzed between each group. All detected circulating miRNAs were examined by ROC analysis and then LASSO dimension reduction to obtain an optimal panel for diagnosis of AMI. A five-year period follow-up towards the AMI and reperfusion patients was performed, and the prognostic value of circulating miRNAs in these patients was estimated by using the Cox regression model, ROC and Kaplan-Meier curves.

**Results:**

Comprehensive global differences of miRNAs transcriptome among AMI, reperfusion patients and healthy controls were identified. A total of 40 miRNAs, called high diagnostic performance miRNAs, including several previous well-studied miRNAs with AUC greater than 0.85 were shown to discriminate AMI with healthy controls. In addition, 29 miRNAs were analyzed to be strongly correlated with the plasma cTnI level, of which 20 overlapped with high diagnostic performance miRNAs. These overlapped miRNAs are over-represented in the pathways which actually reflect the pathological cause of myocardial infarction, as well as the regulation of gene expression and energetic pathway of cellular response to hypoxia. Finally, two miRNAs were analyzed to be significantly correlated to all-cause mortality.

**Conclusion:**

This is the first time to survey plasma miRNAs for the development of AMI diagnostic and prognostic biomarkers at the transcriptome level. A subset of miRNAs exhibited potential diagnostic and prognostic merits for AMI.

**Supplementary Information:**

The online version contains supplementary material available at 10.1186/s40364-024-00690-x.

## Background

Acute myocardial infarction (AMI) is the most serious cardiovascular disease that has been the leading cause of human mortality and morbidity worldwide [1]. It is pathologically caused by disruption of a vulnerable atherosclerotic plaque or erosion of the coronary artery endothelium. AMI is traditionally divided into two large categories: ST-segment elevated myocardial infarction (STEMI) and Non-ST-segment elevated myocardial infarction (NSTEMI), and a small category: unstable angina pectoris which has the similar symptoms as NSTEMI [2]. An earlier accurate diagnosis of AMI using stand-alone or multiple combined approaches may provide believable evidence for initiation of reperfusion therapy to reduce harmful events such as cardiac death and heart failure [3]. Biomarkers, especially those specific proteins circulated around the peripheral blood, have played a critical role in diagnosing AMI during the past decades [4]. Although several crucial biomarkers including creatine kinase-MB (CK-MB), serum myoglobin (Myo), cardiac troponin I and T (cTnI, cTnT) have been established and extensively used to diagnose AMI [5, 6], the major weakness of these biomarkers is that they lack enough disease-specificity or their concentrations need a relatively long duration from onset of symptom to a diagnostic level in the peripheral blood [7–9]. Thus, efforts to exploration of novel biomarkers with better time-effectiveness, diagnostic sensitivity and specificity have never been stopped. Using gene expression microarray and ELISA technologies, Park et al. analyzed a cohort of blood transcriptome and evaluated several protein biomarkers such as PGLYRP1, IRAK3 and VNN3 that are more sensitive and specific for diagnosis of early-stage STEMI than traditional CK-MB or troponin [10]. In addition, by combination of four independent studies, Xiao et al. integrated and analyzed gene expression microarray data and found that *FCER1G* and *PTGS2* had a relatively high diagnostic sensitivity for AMI [11]. These results indicated that these proteins have the potential to serve as putative clinical signatures in AMI diagnosis.

In addition to protein biomarkers, extracellular microRNAs (miRNAs) circulated in the bloodstream are being explored for their potential as biomarkers for cardiovascular diseases [12–24]. The most striking advantage of this class of small RNAs as applicable bio-signature is that they exhibited unexpected stability in the plasma, presumably ascribed to their physical association with microparticles (exosomes, microvesicles, and apoptotic bodies) or RNA-binding proteins (Argonaute2 [Ago2]) or lipoprotein complexes (high-density lipoprotein [HDL]) to prevent to be degraded by the high level of RNase activity in plasma [25–30]. Another important advantage is that elevation of miRNA level in the peripheral blood needs a shorter time than cTnI both in infarcted patients or animals [16]. Several studies have demonstrated that miR-208a, miR-499, miR-1 and miR-133 are consistently elevated in the plasma of AMI patients within a few hours after onset of the myocardial infarction [17–24]. Of these 4 miRNAs, miR-208a is only the “cardiac-specific” miRNA [31], which could not be detected in plasma from healthy control subjects or non-AMI patients with the chest pain symptom using qRT-PCR but was easily detectible in the majority proportion of the AMI patients [4, 16]. Furthermore, the performance of the above four miRNAs in the diagnosis of AMI was evaluated by side-to-side comparisons of receiver operating characteristic (ROC) curves, and miR-208a showed a superior ROC curve to the three other miRNAs [16]. However, conflicting result was also observed for the level of circulating miR-208a which was not detected as elevation in AMI patients [19], suggesting that multiple independent study populations are still required to be replicated to further confirm the diagnostic ability of this promising bio-signature.

Although several miRNA biomarkers have been identified and proposed in the earlier studies, it is keen to know if there are any other unrevealed miRNAs with better performance involved in diagnosis of AMI. Because of the recent absence of holistic surveillance of circulating miRNA (c-miRNA) from healthy, AMI and reperfusion subjects at the transcriptome level in a relatively large cohort, it is urgent to understand the global c-miRNA differences among these populations, as well as the c-miRNA dynamic changes during myocardial infarction and revascularization. It is also interesting to know that is there significant differences of c-miRNA profile between different AMI categories. Once the global differences of c-miRNA profile were acquired, it will be expected that combining multiple differential c-miRNAs into a diagnostic model would provide greater accuracy than a single miRNA. In the present study, using next-generation sequencing (NGS) technology, we profiled c-miRNA landscapes in the plasma of healthy controls, AMI patients and the AMI patients who were subjected to reperfusion therapy. We analyzed the differences between groups and further identified a catalog of miRNAs that have the potential for exploration of novel biomarkers for the diagnosis of AMI. We also conducted a five-year period follow-up towards the AMI and reperfusion patients and analyzed all c-miRNA levels correlated to the overall survival (OS). We envisage these novel miRNAs could be further assessed by independent population study and provide valuable resource for diagnosis and prognosis of AMI.

## Methods

### Population, cohort classification and time-point of sample collection

The population enrolled in this study are consecutive those who visited the Department of Cardiology (or Chest Pain Center) in Baotou Central Hospital with the symptom of “chest discomfort” from November 2018 to April 2019 for the discovery cohort, and September 2021 for the validation cohort. According to the study design, the discovery cohort enrolled three kinds of populations, including healthy control subjects (control group), AMI patients (AMI group) and AMI patients undergoing revascularization that were treated by percutaneous coronary interventions (PCI, reperfusion group), whereas the validation cohort only enrolled AMI patients and healthy controls. The inclusion criteria for AMI patients were based on the expert consensus of the third universal definition of myocardial infarction [32]. Briefly, patients with AMI were clinically diagnosed by typical acute chest pain, biochemical markers cTnI, electrocardiogram change and/or coronary angiography, and were further subdivided into STEMI and NSTEMI subgroups. Only AMI patients from the onset of the first symptom to emergency within 48 h were included for further miRNA profiling. Myocardial infarcted patients were excluded if they had previously received intravenous thrombolytic or anticoagulant therapy. Healthy controls were referred to the individuals who were diagnosed with normal electrocardiogram, normal cTnI level and no history of severe or uncontrolled medical illness, malignancy and infectious diseases. In the discovery cohort, we gathered and assessed the c-miRNA profiles of 91 subjects for biomarker discovery, including 54 AMI patients (32 STEMI and 22 NSTEMI), 17 healthy controls and 20 STEMI patients who were treated by PCI. Of these 20 STEMI patients whose arterial occlusion have restored patency, 12 subjects were the same patients as STEMI subgroups. In the validation cohort, the c-miRNA transcriptomes of 10 AMI patients (5 STEMI and 5 NSTEMI) and 10 healthy controls were assessed for biomarker evaluation. An initial venous blood which was used for parallel measurement of miRNA and cTnI was sampled after the patients visited the hospital (AMI and control groups), and the blood sample from the reperfusion group for miRNA profiling was collected 48 h later after PCI. Plasma used for miRNA profiling was obtained by two-step centrifugation from the peripheral blood and the supernatant was harvested for further operation. Plasma cTnI level was determined by a commercially available chemiluminescent immunoassay kit (Hotgen, Beijing, China).

### RNA preparation, library construction, and NGS sequencing

Total RNA was extracted from about 2mL of each plasma sample using a commercially available miRNeasy Micro Kit (Qiagen, Germantown, MD, USA) according to the manufacturer’s instructions. RNA quality and concentration from each extract were assessed by using an Agilent 2100 Bioanalyzer (Agilent, Santa Clara, CA, USA). Approximately 200 ng of total RNA from each sample was used for library construction. Small RNA library preparation was performed with the TruSeq small RNA Sample Prep Kit (Illumina, San Diego, CA, USA) according to the instructions. Briefly, 3’ and 5’ specific adapters were ligated onto the RNA 3’ and 5’ ends by using T4 RNA ligase, respectively. Then, reverse transcription followed by eleven cycle PCR was applied to create cDNA constructs based on the adapters-ligated RNA as the templates. The amplified cDNA was separated by 6% polyacrylamide gel electrophoresis (PAGE), and the target (~ 150nt small cDNA constructs which contain both 5’ and 3’ adapters) was retrieved and recovered by using the gel extraction method, and was subsequently concentrated by ethanol precipitation. The quality control of the small RNA library was validated by Agilent High Sensitivity DNA Kit (Agilent, Santa Clara, CA, USA) using Agilent 2100 Bioanalyzer. Total concentration of the library was quantified through Quant-iT PicoGreen dsDNA Assay Kit (Invitrogen, Wilmington, DE, USA) using a Quantifluor-ST fluorometer (Promega, Madison, WI, USA). Finally, the valid concentration of the library was determined via the qPCR method by using a StepOnePlus Real-Time PCR Systems (Applied Biosystems, Waltham, MA, USA). Single-end sequencing with a 50-bp strategy was performed at a depth of 20 M for one sample/lane in the Illumina HiSeq2500 platform (Personal Biotechnology, Shanghai, China).

### Quantification and differential analyses of circulating miRNA among groups

The high-quality reads generated by NGS technology were first aligned to the human genome (hg19), then annotation and quantification of known mature miRNAs in each subject were conducted by realigning those genome-aligned reads against miRbase 22.1 using the miRDeep2 package [33]. DESeq2 package in R software was used to normalize the miRNA level among each population for different library sizes, and to assess differentially-circulated miRNAs by group or subgroup comparisons including AMI versus control, reperfusion versus control, AMI versus reperfusion, and STEMI versus NSTEMI. Differentially-circulated miRNAs were filtered with the criteria by both an adjusted *P* value less than 0.05 and a change in expression levels of at least twice (|log_2_FoldChange|>1) between groups or subgroups. Unsupervised hierarchical clustering of the expression pattern of differentially-circulated miRNAs between groups was conducted using the pheatmap package.

### Development and functional analyses of diagnostic miRNA associated with AMI

To development of potential diagnostic miRNA associated with AMI, AUC (the area under the receiver operating characteristic curve) of all detected circulating miRNAs was calculated using the pROC package, and the ROC (receiver operating characteristic) curves of selected miRNA were plotted to compare the efficiency of these diagnostic markers. The best cut-off values corresponding to the best sum of sensitivity and specificity in ROC curves were obtained to distinguish AMI and healthy control subjects in a confusion table. The number of these differentially expressed circulating miRNAs was then reduced to screen diagnostic markers by using the least absolute shrinkage and selection operator (LASSO) with the tuning parameters determined by the expected generalization error estimated from 10-fold cross-validation. Common confounders, including age and heart rhythm status, were stratified to examine their impact on model predictions. Games-Howell test was performed to compare the prediction score between AMI and control groups in each of the two age bins or two kinds of heart rhythm bins. The relationships between all circulating miRNAs and cTnI levels were analyzed by Spearman’s correlation test. Both a *P* value less than 0.05 and a correlation coefficient greater than 0.40 were considered to be significantly correlated. Kyoto Encyclopedia of Genes and Genomes (KEGG) enrichment analysis for circulating miRNAs was performed by using miEAA2.0 [34]. The significantly over-represented KEGG pathways were selected as *P* < 0.01.

### Identification of miRNAs associated with overall survival of AMI

All patients with AMI and reperfusion in this study received long-term follow-up, and were contacted by interview or telephone at 30 and 60 months after hospital discharge. The primary end-point was all-cause mortality during follow-up period. The univariate Cox regression analysis was performed to identify miRNAs associated with survival. The significant miRNAs identified by Cox regression were subjected to calculating the AUC value of time-dependent ROC curve from censored survival data using the timeROC package. Only the miRNAs with AUC greater than 0.75 were retained. The best cut-off points of the above two-step positive miRNAs were obtained using the “surv_cutpoint” function (survminer package) to discriminate patients into different prognostic groups. Except that hsa-miR-6875-5p and hsa-miR-7855-5p use the median as cut-off points because they failed to implement this method successfully. Kaplan-Meier curves were then plotted to evaluate the correlations between miRNAs and overall survival. Hazard ratio (HR) and *P* values were computed by using the “hazard.ratio” function in the survcomp package. Multivariate Cox regression was conducted on the selected miRNAs and reperfusion status to explore the independent risk factors for AMI prognosis. All aforementioned *P* values were two-sided.

## Results

### Profiling of differentially circulated miRNAs and their diagnostic performance among AMI, control and reperfusion subjects

**Fig. 1:**
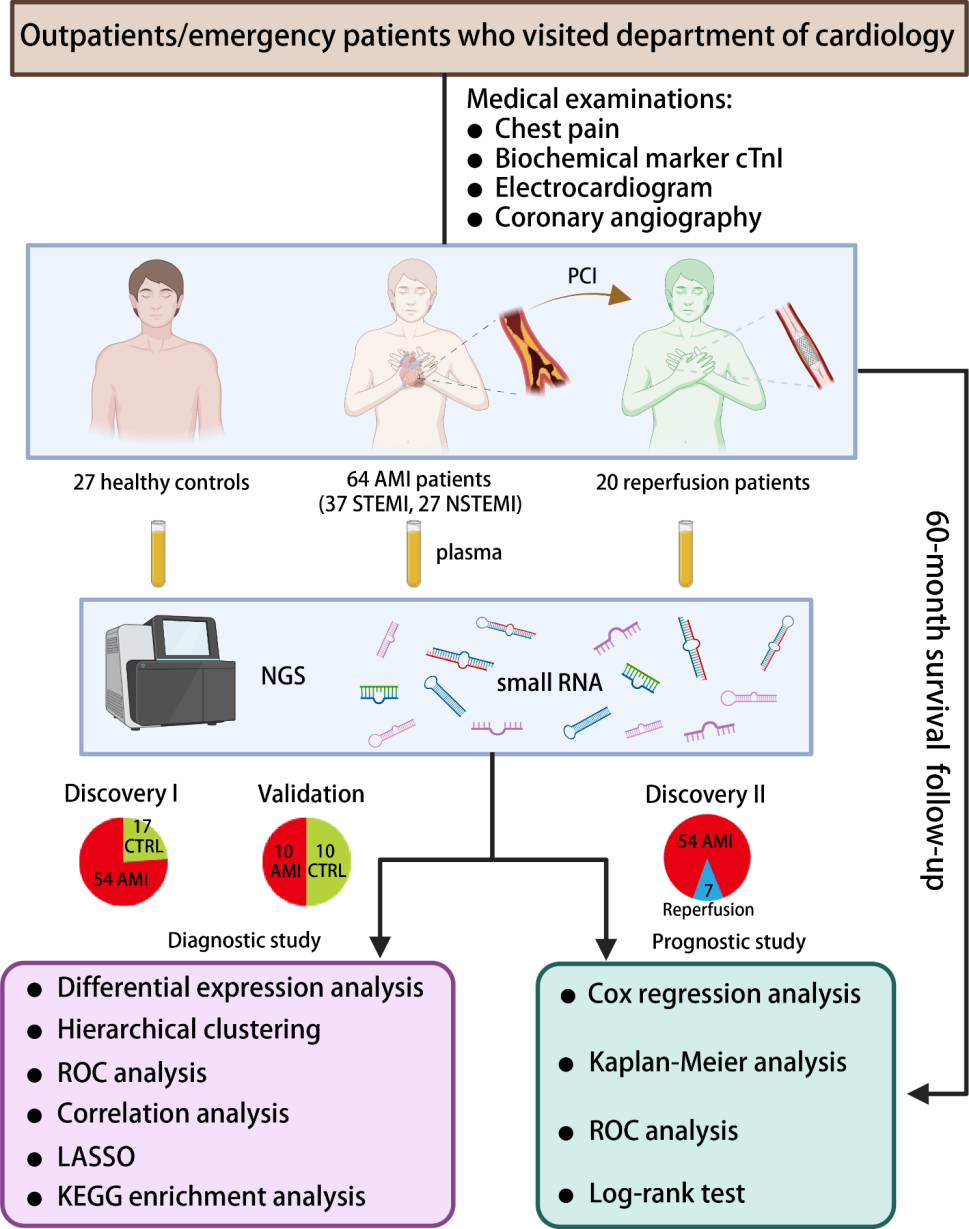
The workflow chart of this study. A total of 111 research subjects were enrolled in this study. The two cohorts for diagnostic biomarker development were designated Discovery I and validation cohort, while the cohort for prognostic marker research was designated Discovery II

Based on the inclusion criteria, we collected a total of 111 research subjects and divided them into two cohorts according to the sampling periods, the biomarker discovery (91 subjects) and validation (20 subjects) cohorts. Then, we performed small RNA transcriptome sequencing for each of these subjects by using NGS technology, and the cTnI levels of AMI patients and healthy controls from two cohorts were parallel investigated. The overall work flow of this study is described in Fig. 1.

**Fig. 2:**
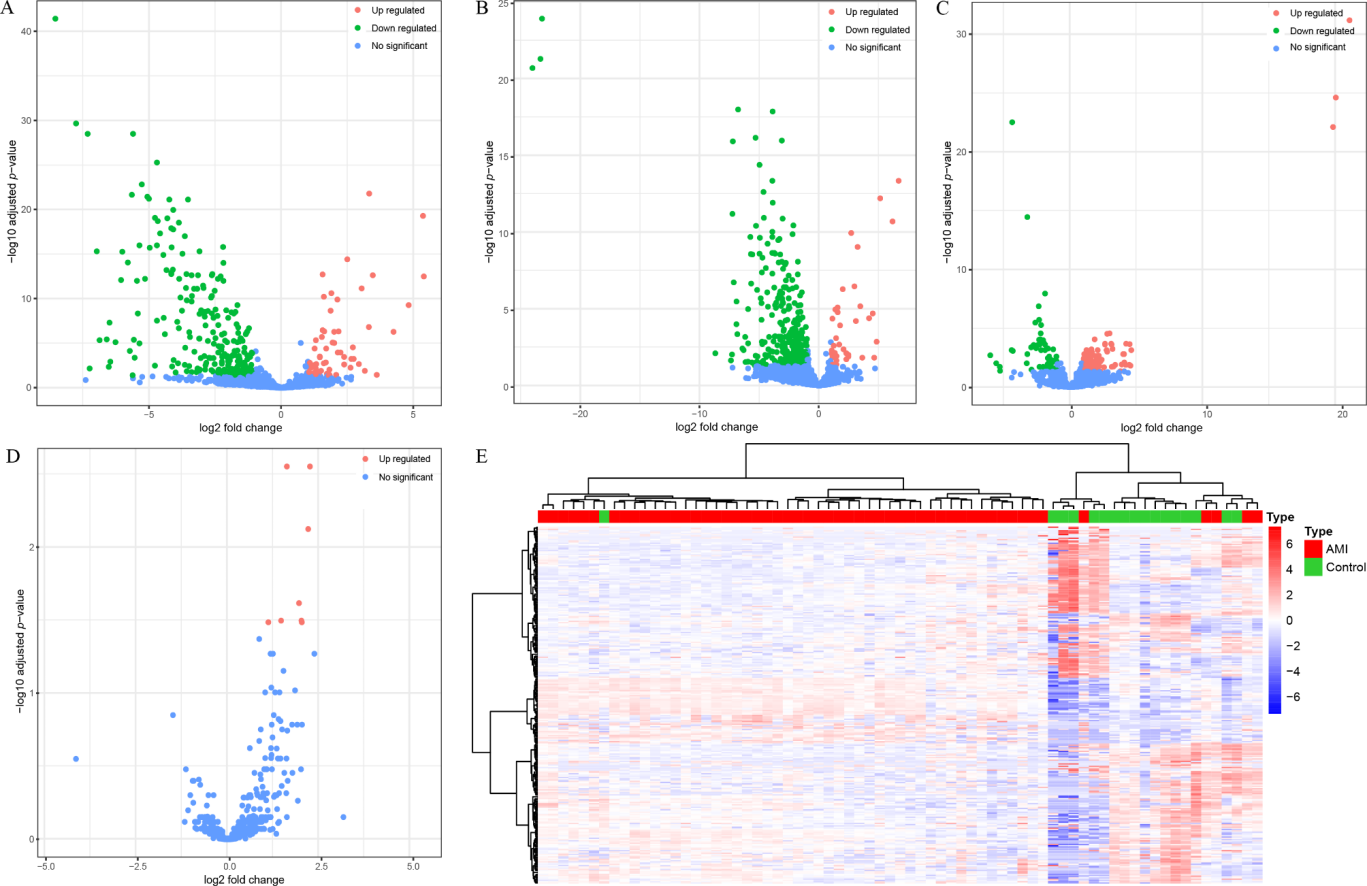
Identification of global differences of c-miRNA between groups or subgroups. Volcano plot of differentially-circulated miRNAs between (**A**) AMI and healthy control (**B**) reperfusion and healthy control (**C**) AMI and reperfusion (**D**) STEMI and NSTEMI. Red, green and blue color-label pellet indicated up-regulation, down-regulation and no significant change of circulating miRNA level in the former group/subgroup compared to the latter group/subgroup. (**E**) Unsupervised hierarchical clustering and heatmap of 54 AMI patients and 17 healthy individuals based on the 288 dif-c-miRNA between AMI and healthy control

To discover high quality miRNA biomarkers, the most important thing is to understand the global alternations of c-miRNA transcriptome among healthy, myocardial infarcted and therapeutic individuals. We then conducted differential analysis among these groups in the discovery cohorts. The baseline characteristics of 91 subjects in the discovery cohort were shown in Table 1. High-throughput sequencing generated an average of 20.38 million high quality reads for each population, and the distribution of reads quantity for each group was described in Figure S1A. After realigned transcriptome data on the miRBase, a total of 1659 known mature miRNAs could be detected at least in one of 91 subjects of plasma. The top 40 c-miRNAs (mean with 95%CI, *n* = 91) were described as Figure S1B with the miR-486-5p, miR-451a, and miR-320a-3p being the top three abundant miRNAs. We then performed the differentially-circulated miRNAs (dif-c-miRNAs) analyses between groups or subgroups, including AMI and healthy control, reperfusion and healthy control, AMI and reperfusion, as well as STEMI and NSTEMI. Based on our criteria, we identified 288 dif-c-miRNAs between AMI patients and healthy controls, consisting of 58 up-regulated and 230 down-regulated c-miRNA in AMI patients compared with control subjects (Fig. 2A), which can serve as potential candidates for further exploration of novel miRNA biomarkers for myocardial infarction diagnosis. It is worth noting that previously well-documented miRNA biomarkers, such as miR-208a-3p, miR-208b-3p, miR-499a-5p, miR-1-3p and miR-133b have been completely included in the 58 up-regulated c-miRNAs in AMI patients compared to the healthy control. The difference appears to be slightly changed after reperfusion treatment, reserving 37 up-regulated and 294 down-regulated c-miRNAs compared to the healthy individuals (Fig. 2B). There was still obviously a global difference between AMI and reperfusion groups (Fig. 2C), but this difference was remarkably smaller than the former two group comparisons. Merely eight up-regulated but no down-regulated c-miRNAs were detected between STEMI and NSTEMI (Fig. 2D), suggesting that the expression patterns of c-miRNA in these two pathological categories are quite similar.

**Table 1:** Baseline demographic characteristics of the discovery cohort

Characteristics	STEMI (*n* = 32)	NSTEMI (*n* = 22)	Reperfusion (*n* = 20)	Control (*n* = 17)	*P*-value
Age (years)	59.03 ± 10.23	66.59 ± 10.5	60.4 ± 7.83	56.71 ± 9.14	0.010
Sex (male) (%)	28 (87.5)	11 (50)	16 (80)	8 (47.06)	0.003
Diabetes mellitus (%)	12 (37.5)	13 (59.09)	5 (25)	4 (23.53)	0.075
Hypertension (%)	11 (34.38)	18 (81.82)	6 (30)	9 (52.94)	0.001
Hyperlipemia (%)	13 (40.63)	12 (54.55)	11 (55)	13 (76.47)	0.125
Reperfusion (%)	29 (90.63)	5 (22.73)	20 (100)	0 (0)	< 0.001
Arrythmia (%)	17 (53.13)	9 (40.91)	12 (60)	8 (47.06)	0.643
Blood glucose (mmol/L)	8.3 ± 3.27	9.06 ± 4.67	7.83 ± 2.96	6.56 ± 1.64	0.263
Diastolic pressure (mmHg)	79.5 ± 15.07	85.91 ± 10.54	86.25 ± 27.76	87.06 ± 15.21	0.209
Systolic pressure (mmHg)	125.91 ± 25.36	141.82 ± 21.96	133.25 ± 34.19	138.82 ± 24.97	0.101
Total cholesterol (mmol/L)	4.83 ± 0.91	4.86 ± 1.19	5.19 ± 0.87	4.86 ± 0.94	0.587
Triglyceride (mmol/L)	2.31 ± 1.84	2.28 ± 1.3	2.61 ± 1.98	2.52 ± 1.45	0.627
HDL (mmol/L)	1.23 ± 0.36	1.25 ± 0.23	1.18 ± 0.25	1.25 ± 0.32	0.792
LDL (mmol/L)	2.37 ± 0.73	2.53 ± 0.81	2.59 ± 0.59	2.56 ± 0.77	0.697
Creatinine (µmoI/L)	72 ± 2.83	63.13 ± 3.69	70.87 ± 4.27	57 ± 4.24	0.531
cTnl (ng/mL)	15.36 ± 15.23	2.6 ± 2.31	-	0.07 ± 0.03	< 0.001

Data are expressed as the mean ± SD or number of patients (percentages). Continuous variables were compared by using ANOVA or the Kruskal-Wallis H Test, and categorical variables were compared by using the Fisher’s Exact Test between the four groups

Unsupervised hierarchical clustering of the above four comparison pairs was then respectively conducted based on the differentially circulated miRNA patterns from each of the comparison units. Between AMI and control, the heatmap showed that the majority of the population belonging to the same group could cluster together (Fig. 2E). However, the discriminative capacity of differentially circulated miRNA patterns declined in AMI versus reperfusion (Figure S2A), reperfusion versus control (Figure S2B), and entirely failed to distinguish STEMI and NSTEMI (Figure S2C), which was represented by the extensively intersected distribution of two electrophysiological patients in the clustering map. Besides, it also failed to distinguish three groups using the aggregative dif-c-miRNAs dataset (Figure S2B).

### Diagnostic capacity and dynamic changes of circulating miRNAs during ischemia-reperfusion

**Fig. 3:**
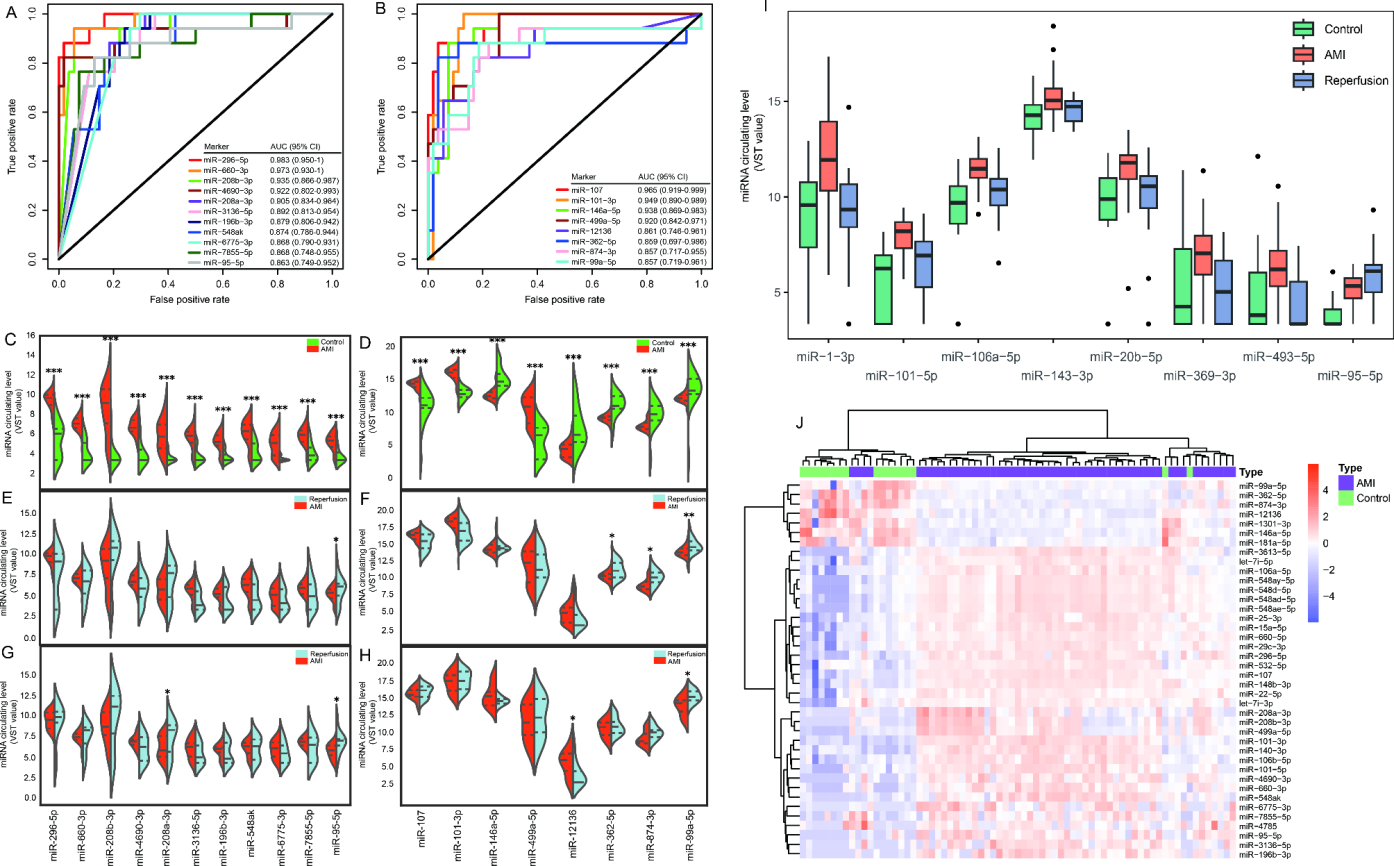
Diagnostic performance and dynamic changes of promising c-miRNAs. ROC curves of 11 low (**A**) and 8 high (**B**) level HDP-c-miRNAs for diagnosis of AMI. Distributions of 19 HDP-c-miRNAs levels are represented by violin plots, and the dashed lines indicate the quartiles. Distribution of 11 low level HDP-c-miRNAs between (**C**) 54 AMI and 17 healthy controls (**E**) 54 AMI and 20 reperfusion patients (**G**) 12 AMI and the same patients who were treated by PCI (paired-sample comparison). Distribution of 8 high level HDP-c-miRNAs between (**D**) 54 AMI and 17 healthy controls (**F**) 54 AMI and 20 reperfusion patients (**H**) 12 AMI and the same patients who were treated by PCI (^*^P < 0.05, ^**^P < 0.01, ^***^P < 0.001). (**I**) Dynamic changes of 8 HDP-c-miRNAs with significant up-regulation during myocardial infarction and significant up- or down-regulation during reperfusion. The median and quartiles of each miRNA were shown in the box. (**J**) Unsupervised hierarchical clustering and heatmap of 17 healthy controls and 54 AMI patients based on the 40 HDP-c-miRNAs

Recently, the most excellent biomarker for diagnosis of AMI is cardiac troponin which was extensively spread from the myocardial cell into the bloodstream after myocardial infarction but it circulated extremely low to nearly an undetectable level in the normal individuals. To explore c-miRNA markers for diagnosing AMI, all detected 1659 c-miRNAs were examined by ROC analysis to side-to-side compare the diagnostic sensitivity and specificity. As a result, a total of 40 c-miRNAs with AUC greater than 0.85 were obtained, including 32 up-regulated and 8 down-regulated c-miRNAs in AMI compared to the healthy control group (Table S1). We tentatively termed these 40 miRNAs as high diagnostic performance c-miRNAs (HDP-c-miRNAs). Of these 40 HDP-c-miRNAs, 38 c-miRNAs are involved in the 288 dif-c-miRNAs. The two other HDP-c-miRNAs include let-7i-5p and miR-4785 which have not been detected as significant dif-c-miRNAs between AMI and healthy control group. We firstly focused on those HDP-c-miRNAs that were at least 5-fold up-regulation with theoretical and practical applications for diagnosing AMI, especially those cardiac-specific miRNAs that distributed at a relatively low level in healthy individuals but were significantly up-regulated in AMI patients. It is exciting to find that a single HDP-c-miRNA displaying the best diagnostic performance is miR-296-5p of which AUC reached 0.983 with a 95% confidential interval ranging from 0.95 to 1.00. The average circulating level of miR-296-5p in the AMI group is about 12.6-fold higher than that in the healthy control group. Furthermore, there are other 10 HDP-c-miRNAs, including miR-660-3p, miR-208b-3p, miR-4690-3p, miR-208a-3p [31], miR-3136-5p, miR-196b-3p, miR-548ak, miR-6775-3p, miR-7855-5p and miR-95-5p, also showing low level in the healthy plasma and displaying robust diagnostic performance for AMI (Fig. 3A and C). In addition to low level c-miRNAs, some of the c-miRNAs were shown to perform well in the diagnosis of AMI, such as miR-107, miR-101-3p, miR-499a-5p, and dispersed abundantly both in AMI and control individuals (Fig. 3B and D). Similar high-level circulating patterns were also observed for miR-146a-5p, miR-12,136, miR-362-5p, miR-874-3p and miR-99a-5p (Fig. 3D). In contrast to the former three c-miRNAs, the circulating levels of the latter five c-miRNAs in the plasma were significantly declined in AMI patients compared to control individuals. Therefore, these 19 HDP-c-miRNA with at least 5-fold differences between AMI and healthy individuals are promising candidates for exploration of miRNA biomarkers in diagnosing AMI.

It has been demonstrated that cardiac troponin reached a peak within 24–28 h after myocardial infarction and remained in the circulation for up to 7–10 days [1, 35]. It will be important to understand the dynamic changes of c-miRNAs during myocardial infarction and revascularization. Since the above 19 HDP-c-miRNAs have recently exhibited valuable features in clinical application in this work, we first paid more attention and analyzed the dynamics of 19 HDP-c-miRNAs between AMI and reperfused AMI group by both unpaired (Fig. 3E and F) and paired-sample comparisons (Fig. 3G and H). In general, 17 HDP-c-miRNAs did not show significant differences both at unpaired and paired-sample comparisons between AMI and reperfusion except for miR-95-5p and miR-99a-5p. In addition, the distribution range of most of the low circulating level HDP-c-miRNAs was expanded except for miR-3136-5p and miR-6775-3p after reperfusion therapy, whereas the distribution range of has-miR-146a-5p, a high circulating level HDP-c-miRNA, was obviously narrowed down compared to the AMI group. From both unpaired and paired sample comparisons, consistent elevation was observed for miR-660-3p, miR-208b-3p, miR-208a-3p, miR-95-5p, miR-362-5p and miR-874-3p, while the miR-3136-5p, miR-4690-3p, miR-146a-5p and miR-12,136 were declined after reperfusion therapy. The above results demonstrated that these c-miRNAs may have distinct dynamics in the circulation.

In addition to the 19 HDP-c-miRNA, we also focused on the c-miRNAs with consecutively remarkable dynamic changes during the ischemia-reperfusion period. We analyzed the miRNA transcriptome fluctuations from healthy to myocardial infarction, and then to revascularization status. As results, only one miRNA, miR-95-5p, was detected as consecutive up-regulation in both two processes, and seven miRNAs, including miR-1-3p, miR-493-5p, miR-106a-5p, miR-143-3p, miR-20b-5p, miR-101-5p and miR-369-3p were up-regulated when suffering myocardial infarction, and followed by significant decline after revascularization (Fig. 3I). It is also warranted to emphasize that three of the above eight rapid dynamic c-miRNAs, miR-95-5p, miR-106a-5p and miR-101-5p are involved in the 40 HDP-c-miRNAs. In addition, 17 consecutively down-regulated, and 50 down- followed by up-regulated c-miRNAs during two processes were identified (Table S2). These rapid dynamic c-miRNAs with high diagnostic performance are also promising candidates for further exploration of biomarkers.

The discriminative capacity of 40-HDP-c-miRNAs for AMI and healthy controls was evaluated by unsupervised hierarchical clustering analysis. However, the diagnostic performance of combining 40 c-miRNAs is not robust enough, with several individuals distributed in the wrong category (Fig. 3J). Thus, the development of a combined model with better diagnostic performance is required for practical application.

**Fig. 4:**
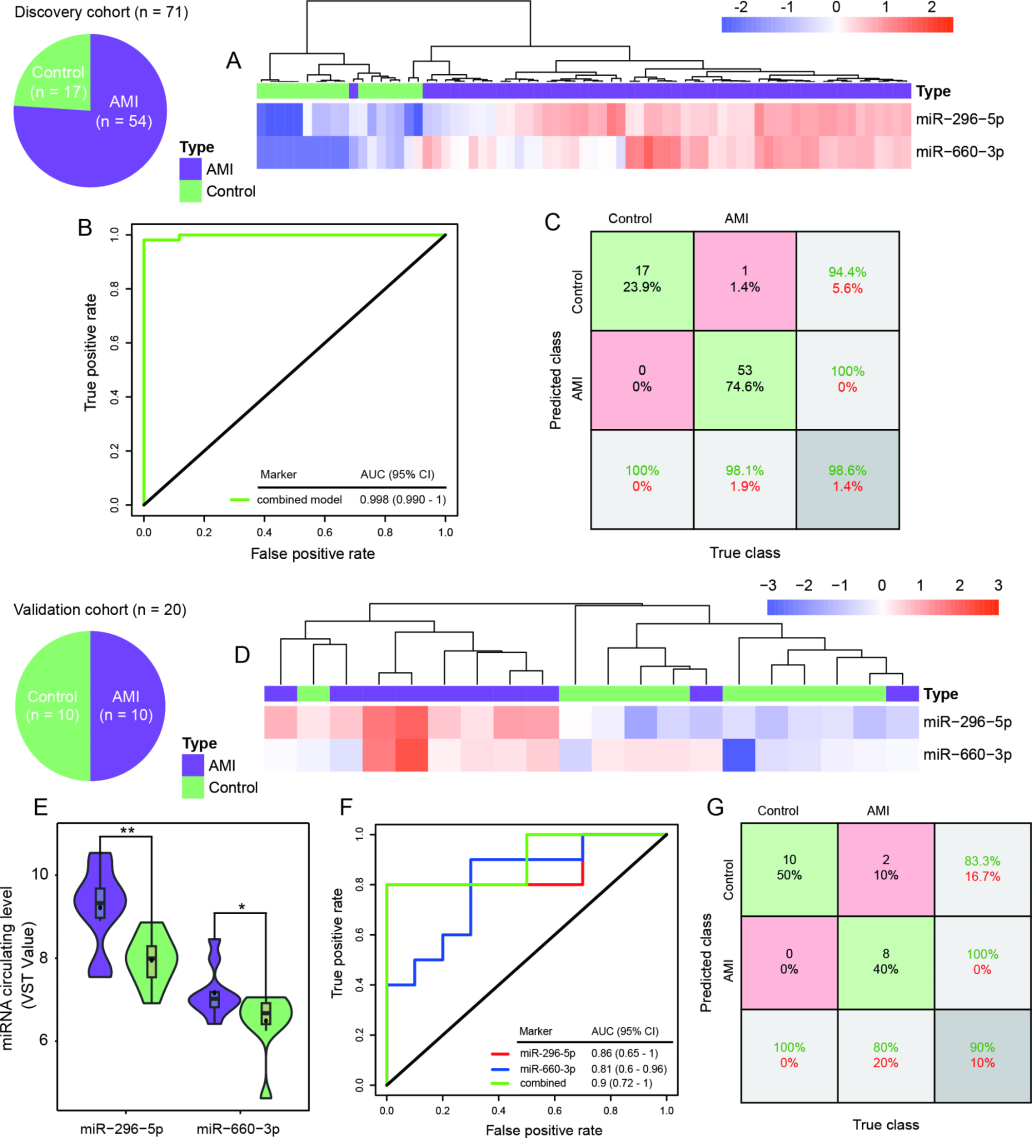
Establishment and validation of a miRNA combining model for AMI diagnosis. Unsupervised hierarchical clustering and heatmap of the patients in the discovery cohort (**A**) and validation cohort (**D**) based on the miR-295-5p and miR-660-3p. The circulating levels of miR-295-5p and miR-660-3p in the validation cohort (^*^P < 0.05, ^**^P < 0.01) (**E**). ROC curve (**B**) and the confusion matrix (**C**) of the combined model in the discovery cohort. ROC curves of two miRNA markers and their combined model in the validation cohort (**F**). The confusion matrix of the combined model (**G**) in the validation cohort. On the confusion matrix plot, the rows represent the predicted class and the columns correspond to the true class. The green cells suggest observations that are correctly classified, while the red cells correspond to incorrectly classified observations. Both the number of observations and the percentage of the total number of observations are shown in each cell

To obtain optimal performance for the diagnosis of AMI, the number of aforementioned 40 HDP-c-miRNAs was reduced by using LASSO. As a result, a panel consisting of two miRNAs was ultimately selected as a diagnostic model, including miR-296-5p and miR-660-3p, which occupied the top two diagnostic performance biomarkers. Unsupervised hierarchical clustering against 54 AMI and 17 control individuals in the discovery cohort was carried out based on the circulating pattern of two miRNAs, and the heatmap revealed that the populations belonging to the same group could be clearly separated except for one AMI subject mixed into the healthy control group (Fig. 4A). The AUC of combining two c-miRNAs reached 0.998 (95%CI = 0.990 to 1.000) which is better than any of a single c-miRNA (Fig. 4B and C). An independent validation cohort consisting of 10 AMI patients and 10 healthy controls was used to further fairly evaluate these two markers. The clustering heatmap showed that two markers can effectively separate two groups of individuals (Fig. 4D). Consistent with the discovery cohort, miR-296-5p and miR-660-3p exhibited higher circulating levels in AMI patients compared to healthy controls in the validation cohort (Fig. 4E). The AUCs of miR-296-5p and miR-660-3p achieved 0.86 and 0.81, respectively, and the AUC of the combined model was 0.90 (Fig. 4F and G).

Several factors such as age and rhythm status (arrhythmia or not) have been reported to affect the accuracy of traditional AMI markers. To further examine possible bias toward the age effects on AMI prediction, we stratified subjects into two age bins of 0 to 60, and 60 to 81 years and compared the model prediction performance on different bins (Figure S3A). Game-Howell test showed there was no significant difference between prediction scores for AMI or control groups in each of the two age bins, and prediction scores were significantly different only in the AMI vs. control group comparisons (all Adjusted *P* value < 0.05) (Figure S3A). The same analysis was also conducted on rhythm status. And the significant difference was only found between AMI and control groups (all Adjusted *P* value < 0.05) (Figure S3B). These further verified that potential confounding effects of age and arrhythmia were excluded from the analysis.

### Diagnostic miRNAs correlated to cardiac troponin

**Fig. 5:**
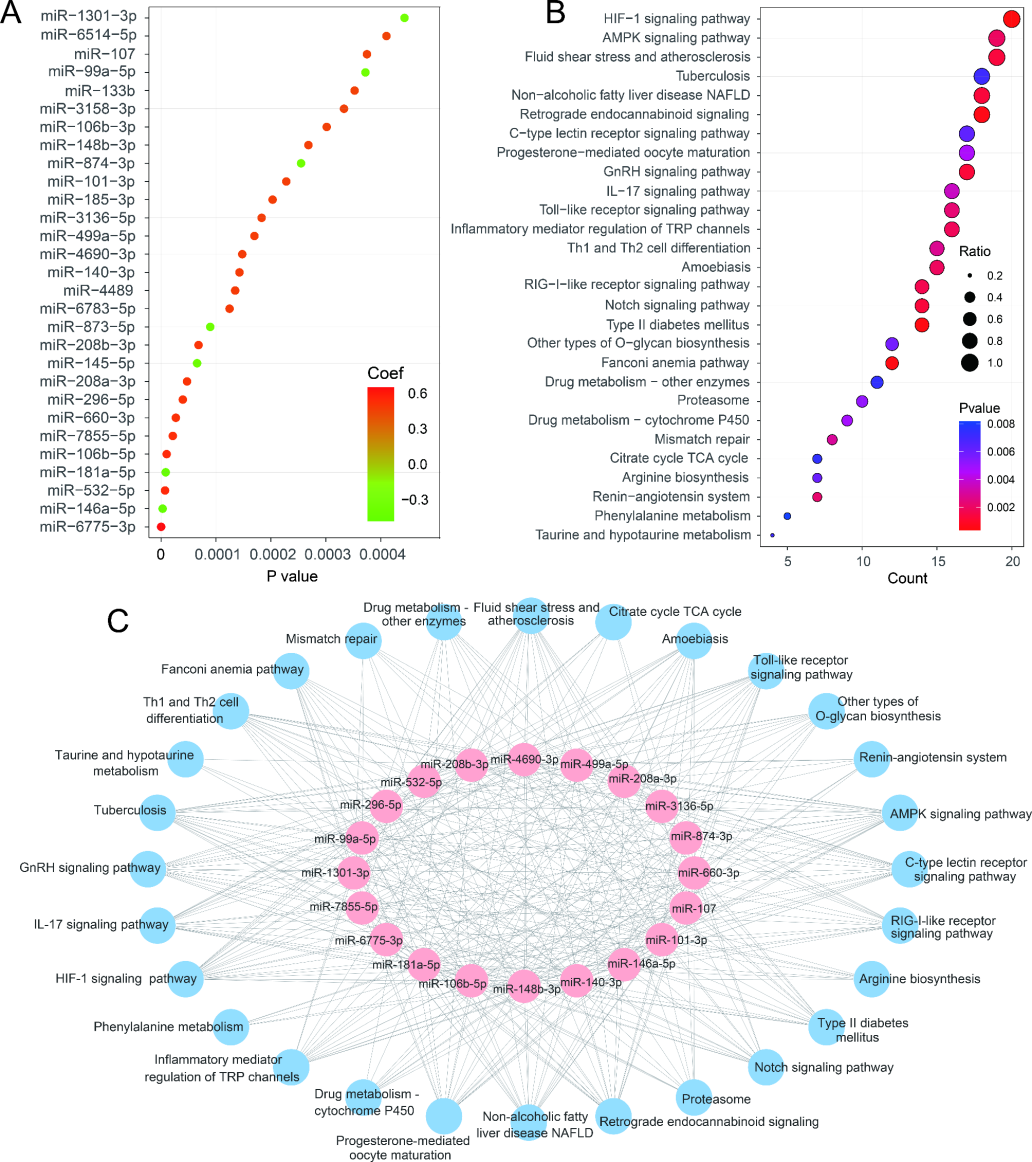
MiRNA-cTnI correlation analysis and pathway mapping, interaction and enrichment of promising miRNAs. (**A**) Dot plot of 29 cTnI-strongly-correlated miRNAs (*P* < 0.05 and coefficient > 0.4). (**B**) KEGG functional enrichment analysis of 20 intersected miRNAs between 29 cTnI-strongly-correlated miRNAs and 40 HDP-c-miRNAs. Only the top 28 significantly enriched pathways (*P* < 0.01) are displayed. (**C**) Interactions of 20 intersected miRNAs with 28 significantly enriched pathways

As cardiac troponin has recently been the “golden standard” biochemical marker for diagnosis of AMI, to determine the c-miRNAs associated with cardiac troponin, c-miRNAs-cTnI correlation analysis was conducted using all 1659 c-miRNAs and cardiac troponin from 54 AMI patients and 17 healthy controls of the discovery cohort. As a result, 29 c-miRNAs were displayed to be strongly correlated with the plasma circulating level of cTnI (*P* < 0.001, |co-efficient| > 0.4), with miR-6775-3p (*P* = 1.97e-8, co-efficient = 0.607) and miR-146a-5p (*P* = 2.77e-6, co-efficient = -0.524) being the top positive and negative correlation c-miRNAs, respectively (Figure S4). We termed these 29 c-miRNAs as cTnI-strongly-correlated c-miRNAs (cTnI-SC-c-miRNAs) (Fig. 5A). Intersectional analysis between 29 cTnI-SC-c-miRNAs and 40 HDP-c-miRNAs showed that 20 cTnI-SC-c-miRNAs overlapped with HDP-c-miRNAs, suggesting that these 20 crossing c-miRNAs are powerful candidates for diagnosing AMI. We termed them as promising miRNAs.

With increased studies involved in the miRNA function and database curation, it is possible to directly annotate the miRNA function without a targeted gene set. Since credible evidence of these 20 c-miRNAs is associated with the accurate diagnosis of AMI and cTnI correlation, we performed KEGG pathway enrichment analysis using 20 intersected c-miRNAs. We found that these 20 intersected c-miRNAs were over-represented in 28 pathways with the top three significant pathways of HIF-1 signaling pathway, AMPK signaling pathway, Fluid shear stress and atherosclerosis (Fig. 5B). The interactions of 28 pathways and 20 miRNAs were shown in Fig. 5C. There were 20, 19, and 19 miRNAs participating in the above three pathways, respectively. The other significantly enriched pathways include Type II diabetes mellitus, Fanconi anemia pathway, Retrograde endocannabinoid signaling, GnRH signaling pathway, and Non-alcoholic fatty liver disease (NAFLD) which seems to be the risk factors of myocardial infarction.

### Identification of prognostic miRNAs markers in AMI

**Fig. 6:**
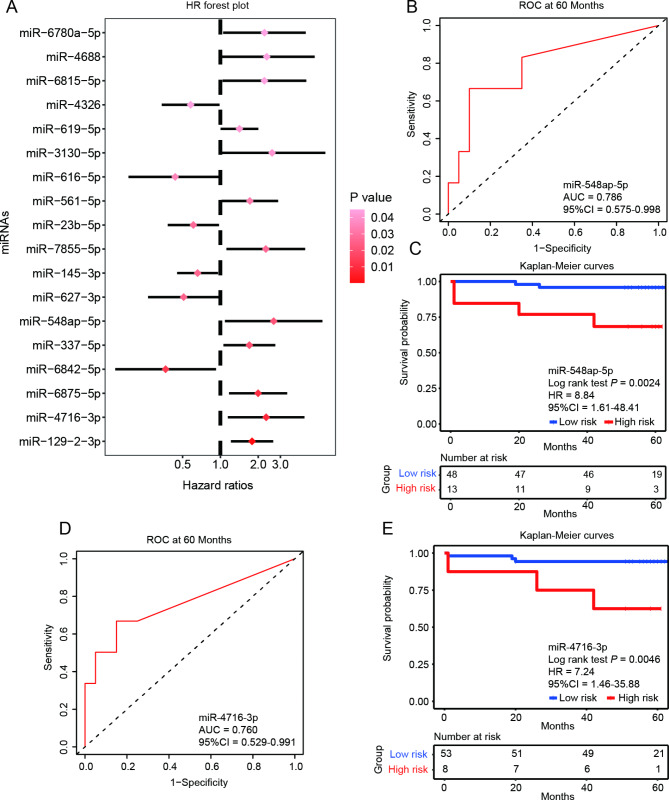
Correlation analysis of miRNAs with OS and prognostic performance of two miRNAs through three-step filtration in the OS prediction in 61 patients. (**A**) Forest plots of hazard ratios of 18 miRNAs in 61 patients. The ROC curves of miR-548ap-5p (**B**) and miR-4716-3p (**D**) were generated for the 60 months OS predictions in AMI patients. The best cut-off value (4.64 for miR-548ap-5p and 5.13 for miR-4716-3p) of the 60-month OS prediction was obtained to divide the patients into low- and high-risk groups. Kaplan-Meier curves of miR-548ap-5p (**C**) and miR-4716-3p (**E**) based on these low- and high-risk groups were plotted to analyze the correlations between two miRNAs and the OS

To exploration of miRNA markers associated with the prognosis of AMI, 62 patients were enrolled in a 60-month period follow-up study, including 54 AMI patients regardless of treatment or not, and 8 reperfusion patients, and the other 12 reperfusion patients are the same ones who come from the AMI group. During the follow-up period, one AMI patient was out of contact, and six (9.84%) patients died. Thus, 61 patients were finally enrolled in the prognostic analysis. We first used univariate Cox regression analysis to screen all potential miRNAs associated with survival. As results, 18 miRNAs showed statistical significance related to survival (*P* < 0.05). The hazard ratio and statistical significance of 18 miRNAs were displayed in Fig. 6A. We further evaluated the prognostic ability of these 18 miRNAs, and results showed that four miRNAs exhibited AUC greater than 0.75, including miR-548ap-5p (Fig. 6B), miR-4716-3p (Fig. 6D), miR-6875-5p and miR-7855-5p (Figure S5). Then, Kaplan-Meier curves were plotted to inspect the discriminative capacity of four miRNAs between the OS for patients in these two risk groups. Two miRNAs, miR-548ap-5p (*P* = 0.0024, Fig. 6C) and miR-4716-3p (*P* = 0.0046, Fig. 6E), displayed significant correlation to OS, indicating that these two miRNAs are promising candidates for prognosis of AMI.

It has been reported early reperfusion within 6 h of symptom onset of the AMI significantly improves prognosis. To evaluate whether reperfusion impact these two markers, we included two prognostic miRNAs (miR-548ap-5p and miR-4716-3p) and reperfusion status in the multivariate Cox analysis to examine the independent risk factors for AMI prognosis. The multivariate analysis revealed that both miR-548ap-5p (*P* = 0.006) and miR-4716-3p (*P* = 0.014) were independent prognostic factors. However, reperfusion status was not an independent prognostic factor in our analysis (*P* = 0.147), although reperfusion therapy was shown to be associated with a good prognosis (HR < 1, Table S3).

## Discussion

In the present study, using deep NGS technology we identified a catalog of miRNAs that have the potential as practicable biomarkers for AMI diagnosis. To our knowledge, limited studies have been previously done associated with *de novo* profiling of miRNA transcriptomes on diagnosis and prognosis of AMI. A decade ago, Wang et al. profiled c-miRNAs from four healthy people’s peripheral blood plasma using miRNA microarray and about 170 miRNAs were detected in all four samples but unfortunately no AMI patients were parallel investigated [16]. Thus, the characterization of the c-miRNA global difference between AMI and normal subjects was suspended. In another study, Tong and colleagues profiled 14 acute coronary syndrome (11 STEMI and 3 NSTEMI) young patients (below 45 years old) and 14 healthy controls using microRNA sequencing [36]. Although a set of miRNA biomarkers, such as miR-183-5p, miR-134-5p, miR-15a-5p and let-7i-5p, has been discovered to discriminate STEMI/NSETMI patients from healthy populations, previously well-described miRNAs, especially those classical miRNA biomarkers were not recurrent, and the data analysis procedure was quite arguable, which was represented by the abandon of low abundance miRNAs during differential expression analysis. In this study, we profiled a total of 91 miRNA transcriptomes of healthy, AMI and reperfusion individuals, and the miRNA global differences between each group were then monitored. Expectedly, an enormous overall difference was identified between AMI and healthy control groups. However, a sustained striking difference was also observed between AMI and reperfusion groups, suggesting that the miRNA pattern in the circulation of AMI patients underwent rapid dynamic change after revascularization for 48 h. Moreover, despite the distinct electrophysiological representation, very limited difference was detected between STEMI and NSTEMI subgroups, indicating that it would be difficult to explore classifiers that were used to discriminate two subgroups.

As the key repository for the development of miRNA diagnostic biomarkers, we identified a total of 288 dif-c-miRNAs between AMI and healthy control groups. Global surveillance for the diagnostic potential of AMI from 1659 c-miRNAs revealed 40 c-miRNAs with AUC greater than 0.85, 38 of which are involved in 288 dif-c-miRNAs, suggesting that the high credibility of these molecules for potential diagnosis of AMI. In addition, a global correlation analysis between all c-miRNAs and cTnI identified 29 cTnI-strongly-correlated miRNAs, of which 20 are overlapped with HDP-c-miRNAs. These overlapped miRNAs are over-represented in the pathways of HIF-1 (hypoxia-inducible factor 1) signaling pathway, AMPK (AMP-activated protein kinase) signaling pathway, Fluid shear stress and atherosclerosis. Acute myocardial infarction is one of the ischemic diseases, in which the coronary artery becomes occluded resulting in severe tissue hypoxia. During this process, the primary transcriptional response to hypoxic stress is mediated by the HIFs [37]. As its name indicates, HIF is recognized as a master modulator of the transcriptional response to hypoxic stress. Under normal oxygen conditions, the α subunit of HIF, is destructed by E3 ubiquitin ligase due to hydroxylation at specific proline residues. By contrast, non-hydroxylated HIFα is protected from degradation and exerts as a transcriptional regulator to produce gene products to increase oxygen delivery and mediate adaptive responses to oxygen deprivation [38]. Similarly, activation of AMPK under hypoxia has been reported repeatedly, but it is still unclear whether this activation is mediated by the hypoxic stimulus specifically, or if it is a side effect of the metabolic and energetic consequences introduced by hypoxia [39]. The activated AMPK signaling pathway inhibits ATP consumption and improves the utilization efficiency of intracellular nutrients as adaptive responses and thus decelerates the cell damage caused by hypoxia [40]. Fluid shear stress regulates the endothelial gene expression of the arteries and influences the atherosclerosis which is the key pathological factor of the acute myocardial infarction [41]. Almost all overlapped c-miRNAs are over-represented in the above three pathways that actually reflect causative agents of myocardial infarction, as well as the regulation of gene expression and energetic pathway of cellular response to hypoxia.

Expectedly, several previously well-documented miRNA biomarkers, miR-208a-3p, miR-208b-3p, and miR-499a-5p were included in 40 HDP-c-miRNAs. Comparatively, the AUC of miR-208a-3p and miR-499a-5p in this work was 0.905 and 0.920, respectively, which is similar to the earlier studies revealed by Wang et al. [16] (0.965 and 0.822) and Liu et al. [42] (0.72 and 0.88) from the Chinese Han population using qRT-PCR technology. Moreover, the other two classical miRNA biomarkers miR-1-3p and miR-133a-5p in this study also possessed the similar diagnostic performance as previously described (0.789 and 0.779 vs. 0.774 [23] and 0.867 [16], respectively). Consistent with previous studies, the superstar biomarker miR-208a-3p in this work was still shown as one of the most cardiac-specific patterns that dispersed at nearly an undetectable level for most of the cases in healthy population but significantly elevated (average 61.7-fold) in the AMI patients. A similar cardiac-specific pattern was observed for miR-6775-3p, miR-196b-3p and miR-3136-5p (Fig. 3A). However, the AUC values of these three cardiac-specific miRNAs were inferior to the classical biomarker miR-208a-3p. Accidentally, several previously unreported miRNA biomarkers, such as miR-296-5p, miR-660-3p, miR-107 and miR-101-3p, also displayed very robust diagnostic capacity in all our detected miRNAs, and their circulating levels in the plasm exhibited a huge difference (at least 5-fold difference) between AMI patients and healthy population. To obtain the best diagnostic performance for practical utility, a model consisting of two miRNAs, miR-296-5p and miR-660-3p was selected from 40 HDP-c-miRNAs to construct a diagnostic model. This diagnostic model performed well both in discovery cohort and validation cohort, although the performance in the validation cohort declined slightly. Another interesting finding is that the dynamic changes of these HDP-c-miRNA had disparity which was represented by their sustained elevation or elevation followed by a decline in the circulation level during ischemia-reperfusion. However, due to the rare appearance of many HDP-c-miRNA in the previous works, together with the above discussion, the diagnostic miRNAs catalog on myocardial infarction identified by various studies are quite divergent. The reasons for the discrepancy among independent studies are possibly ascribed but not limited to the number of enrolled subjects, sampling time distance to the first symptom of chest pain, varying miRNA extraction protocols, technologies of miRNA detection and data normalization procedures, all of which make a direct comparison of these studies complicated. Altogether, our results which were based on the c-miRNA investigation at the transcriptome level provided clues for future similar studies for their utility on diagnosis of AMI.

In addition to the diagnosis of AMI, many studies strive to seek miRNA biomarkers associated with the prognosis of AMI. Matsumoto et al. identified a subset of circulating miRNAs associated with the cardiac death from a total of 667 miRNAs using microarray [43]. They emphasized that two miRNAs, miR-155 and miR-380 whose levels increased in the serum of AMI patients have been connected to the cardiovascular mortality [43]. The classical diagnostic biomarkers, such as miR-1, miR-133a, miR-133b, miR-208a, miR-208b, and miR-499 were also extensively concerned with their prognostic relevance with AMI [44–48]. Some of these microRNAs, including miR-133a and miR-208b, were associated with a significant rise in all-cause mortality at 6 months following acute coronary syndrome [45], especially miR-208b whose level was higher in patients who died within 1 month [49], but this molecule could not predict long-term mortality. In addition, increasing levels of miR-1, miR-208b and miR-499 in patients who underwent primary angioplasty had a detrimental effect on left ventricular ejection fraction, while miRNA-133a has also been shown to be linked to large infarct areas even after reperfusion [47, 48]. Circulating miR-499-5p levels are associated with 12-month cardiovascular mortality after NSTEMI in elderly patients [46]. However, contradictory results showed that both miR-208b and miR-499 were not significant predictors of mortality, and they were not associated with left ventricular function 4-months after myocardial infarction [44, 50]. Similarly, circulating levels of miR-133a were correlated with myocardial damage after AMI but did not independently predict the occurrence of major adverse cardiovascular events [48]. Due to the relatively low patient number enrolled in this study, we used Cox regression, ROC and Kaplan-Meier curves to screen prognostic relevant molecules concerning mortality. However, no classical miRNAs correlated to mortality, and only two previously unreported miRNAs, miR-548ap-5p and miR-4716-3p, were finally screened out. These two miRNAs were both significantly associated with overall survival and performed well on outcome evaluation. These results indicated that no unarguable miRNAs have recently been associated with the prognosis of myocardial infarction and these molecules need to be further proved.

## Conclusions

In conclusion, in this study we identified c-miRNA global differences pertaining to myocardial infraction at transcriptome level, and developed a set of c-miRNAs to diagnose AMI. These miRNAs are convincing and promising because their discovery was based on unbiased and noiseless high-through sequencing, and multiple filtration in a relatively large cohort. Many of them have been demonstrated to be the classical miRNA biomarkers and they can well-explain the pathological causative of myocardial infarction, as well as transcriptional and energetic response to myocardial ischemia at the cellular level. These molecules provided valuable targets for detection and outcome prediction in acute coronary artery diseases.

## Electronic supplementary material

Below is the link to the electronic supplementary material.

Supplementary Material 1

## Data Availability

All high-quality sequencing data have been submitted to NCBI Sequence Read Archive (SRA) under accessions PRJNA1116798 (discovery cohort) and PRJNA1126658 (validation cohort).

## Abbreviations

miRNA: microRNA
AMI: Acute myocardial infarction
STEMI: ST-segment elevated myocardial infarction
NSTEMI: Non-ST-segment elevated myocardial infarction
ROC: Receiver operating characteristic
AUC: The area under the receiver operating characteristic curve
c-miRNA: Circulating microRNA
dif-c-miRNA: Differentially-circulated microRNA
HDP-c-miRNA: High diagnostic performance circulating microRNA
LASSO: Least absolute shrinkage and selection operator
OS: Overall survival
PCI: Percutaneous coronary interventions
KEGG: Kyoto Encyclopedia of Genes and Genomes
AMPK: AMP-activated protein kinase
