# Supplementary material for: Global surveillance of circulating microRNA for diagnostic and prognostic assessment of acute myocardial infarction based on the plasma small RNA sequencing
Source: Biomark Res. 2024 Nov 19;12:143. doi: 10.1186/s40364-024-00690-x (PMC11577892; doi:10.1186/s40364-024-00690-x)
Supplement: Supplementary file 1 — Supplementary Material 1 [file 40364_2024_690_MOESM1_ESM.docx]

**Supporting Information**

Supporting information in this study includes three supplementary figures and two supplementary tables.

1. Supplementary Figures:


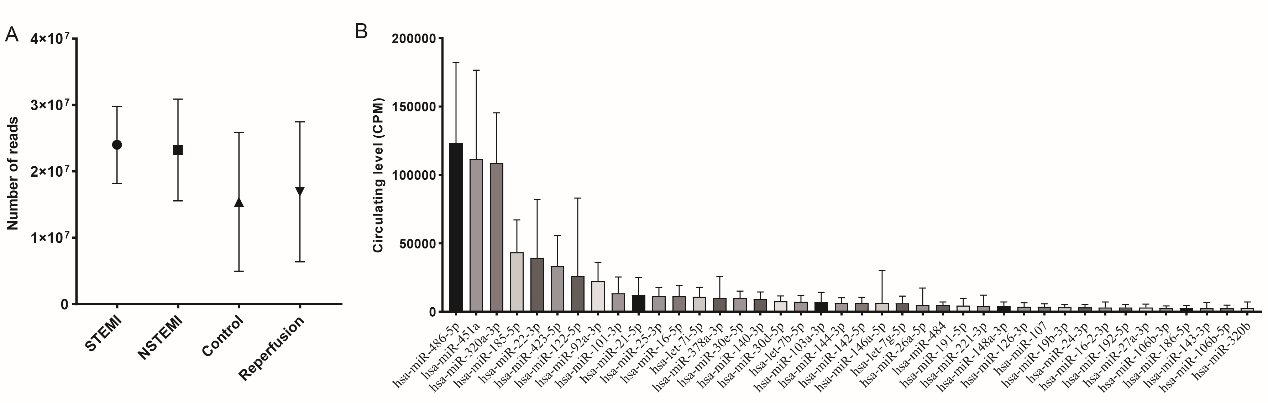


Figure S1. Representations of high-throughput sequencing and miRNA abundance in the circulation. (A) Distribution of NGS high-quality reads in each group or subgroup. (B) Bar plot of top 40 mature miRNAs annotated by combination of all samples. The counts per million (CPM) of each miRNA was calculated. The average plus minus standard deviation of each c-miRNA was shown in the bar.


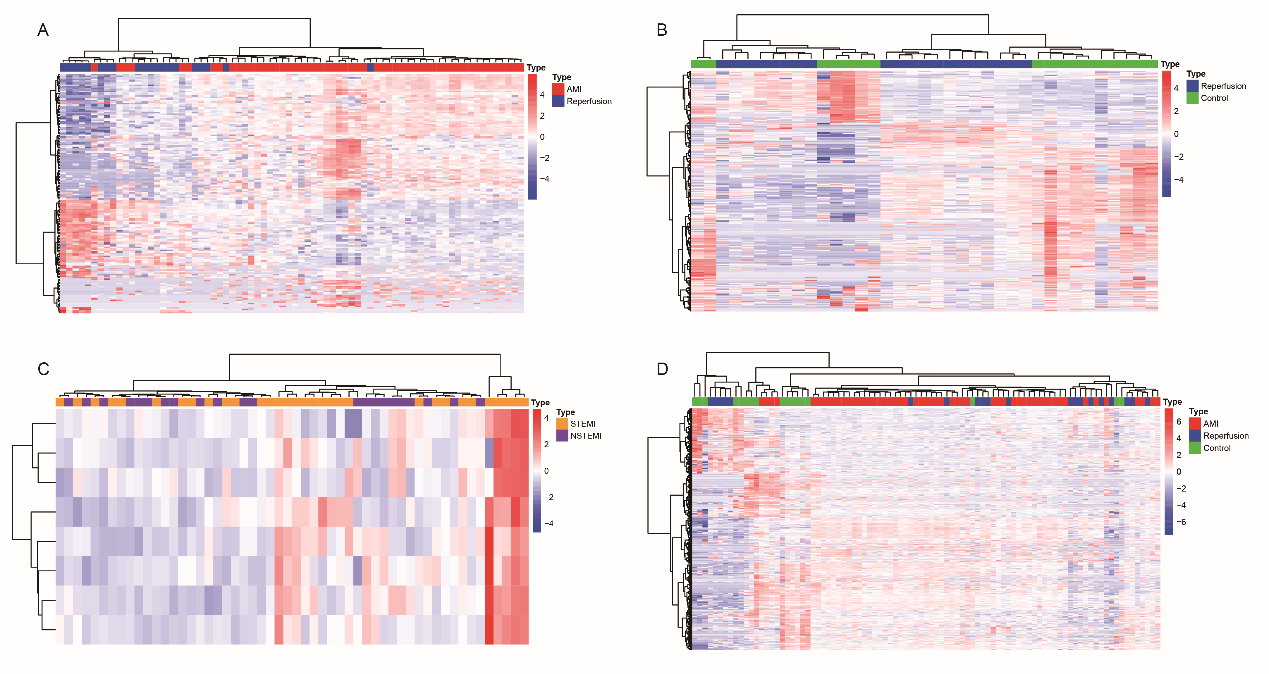


Figure S2. Unsupervised hierarchical clustering and heatmap between groups or subgroups. Differentially-expression analysis identified 158, 331 and 8 dif-c-miRNAs between (A) AMI and reperfusion groups (B) reperfusion and healthy control groups (C) STEMI and NSTEMI subgroups, respectively, and an aggregated 777 dif-c-miRNAs among AMI, reperfusion and healthy control groups. The heatmaps were created by unsupervised hierarchical clustering methods based on the circulating patterns of the above dif-c-miRNA.


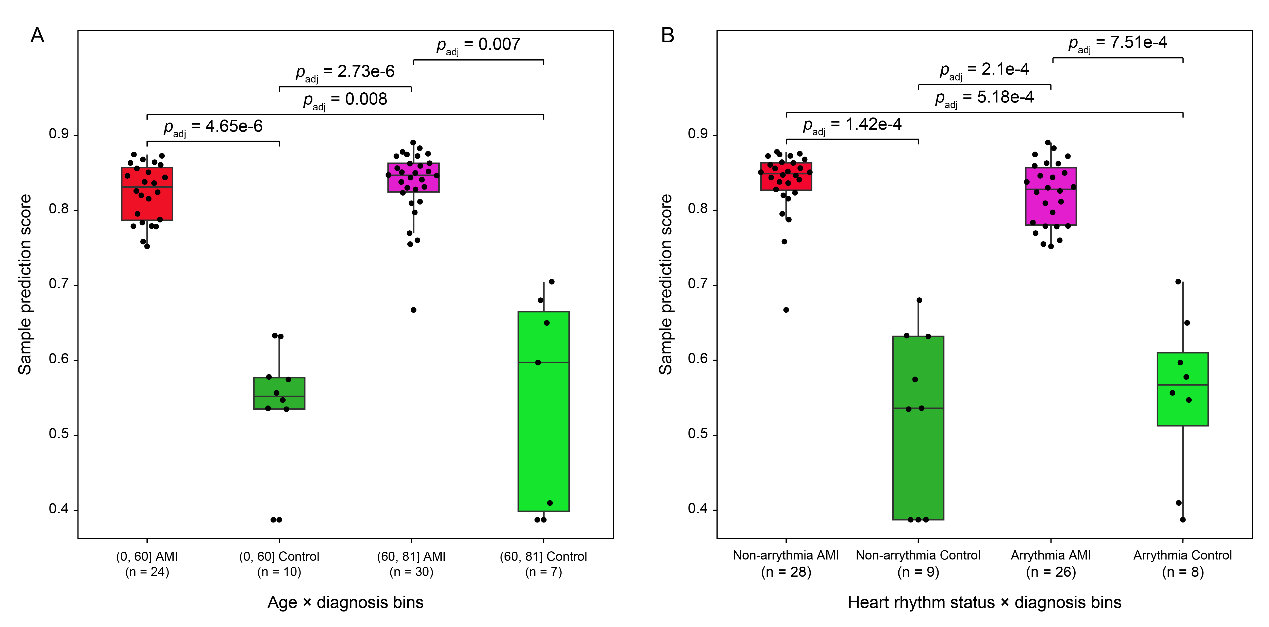


Figure S3. The distribution of prediction scores in stratified analyses of age (A) and heart rhythm status (B). Both analyses divided 71 subjects into 4 groups. Games-Howell tests were performed to compare the group differences and only significant comparisons were shown.


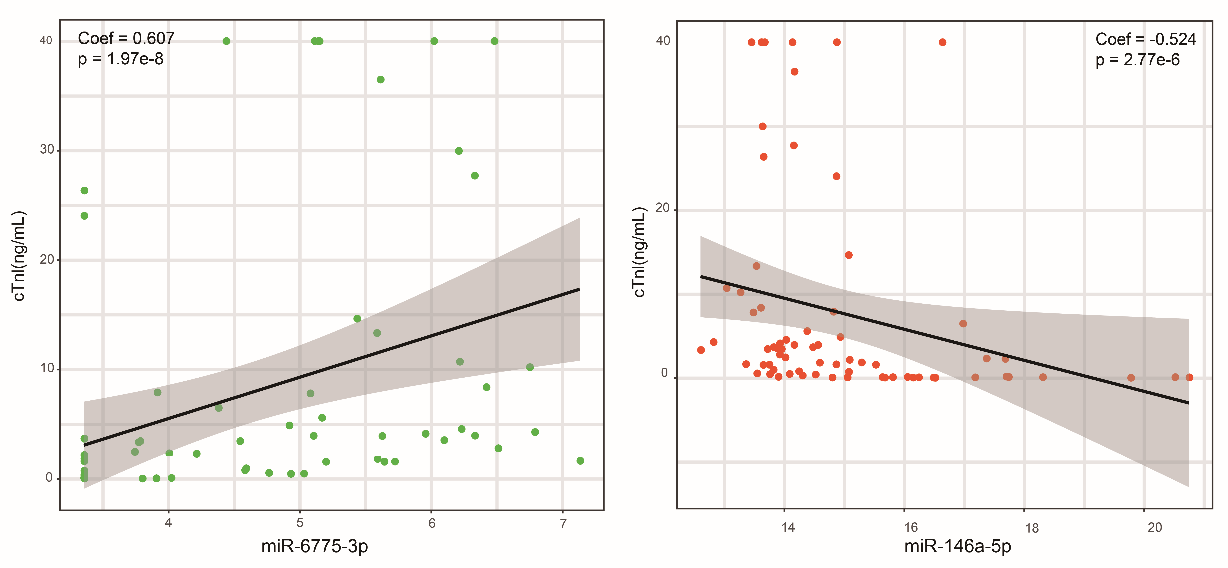


Figure S4. Representations of top positive and negative cTnI-correlated miRNAs.


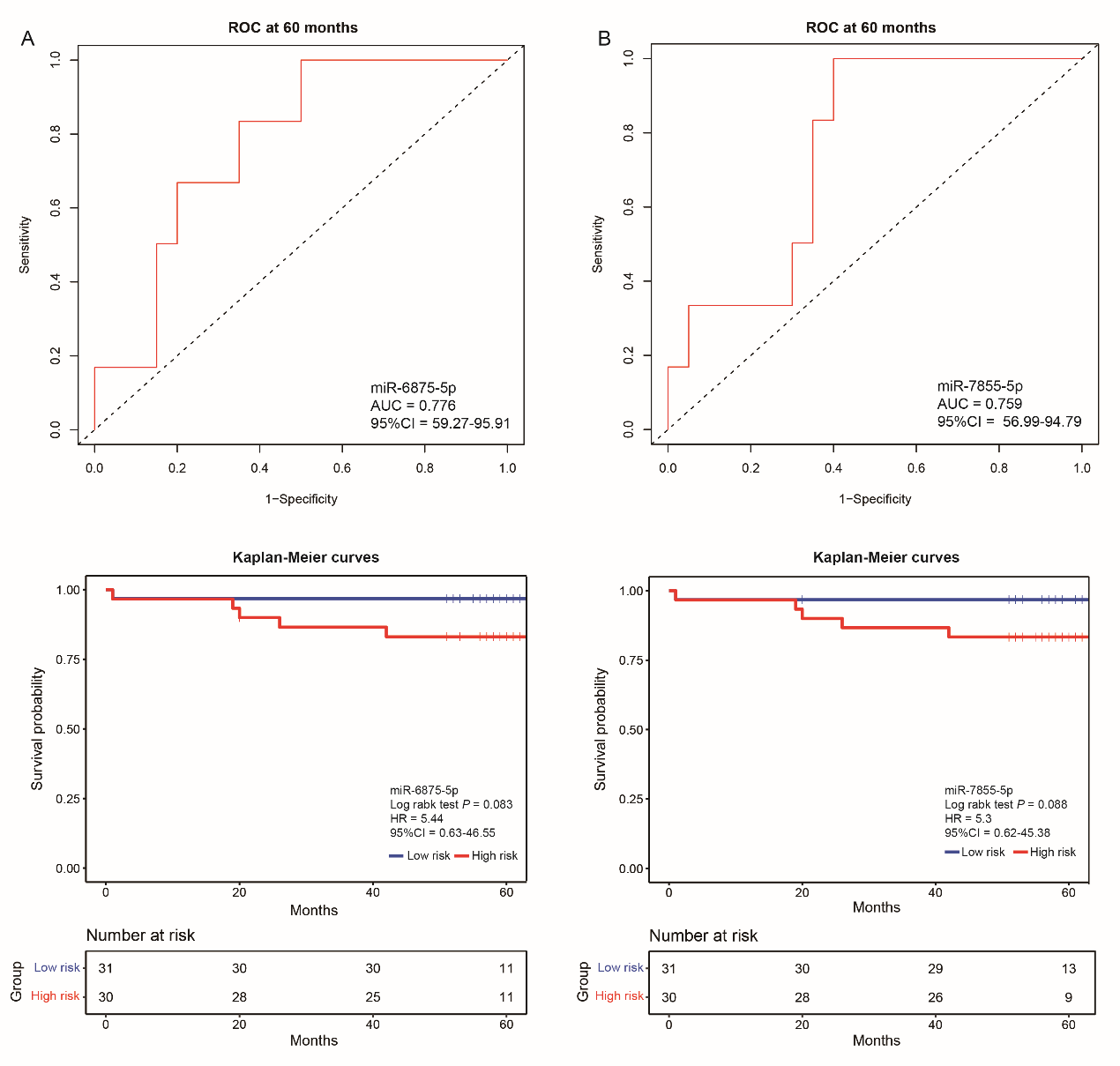


Figure S5. The ROC and Kaplan-Meier curves of (A) miR-6875-5p and (B) miR-7855-5p. Although these two miRNAs both have a higher AUC value (>0.75), they were not significant correlated to OS analyzed by Kaplan-Meier curves.

2. Supplementary Tables:

Table S1. 40 c-miRNAs with AUC greater than 0.85 discriminating AMI with healthy controls

| miRNA | VST value [Median(Q1-Q3)] | | Change | AUC | 95%CI |
| --- | --- | --- | --- | --- | --- |
|  | Control | AMI |  |  |  |
| hsa-miR-296-5p | 6.009(3.354-6.542) | 9.691(8.956-10.004) | Up regulated | 0.983 | 0.951-1 |
| hsa-miR-660-3p | 3.354(3.354-5.091) | 7.063(6.613-7.608) | Up regulated | 0.973 | 0.929-1 |
| hsa-miR-107 | 12.448(12.054-13.711) | 16.115(15.531-16.417) | Up regulated | 0.965 | 0.918-0.998 |
| hsa-miR-101-3p | 14.319(13.845-15.108) | 18.027(17.116-18.433) | Up regulated | 0.949 | 0.891-0.989 |
| hsa-miR-146a-5p | 16.481(15.801-17.733) | 14.016(13.65-14.756) | Down regulated | 0.938 | 0.876-0.984 |
| hsa-miR-532-5p | 11.686(10.834-13.555) | 14.355(14.002-14.614) | Up regulated | 0.938 | 0.869-0.986 |
| hsa-miR-140-3p | 15.933(14.794-16.54) | 17.431(17.019-17.75) | Up regulated | 0.937 | 0.876-0.983 |
| hsa-miR-208b-3p | 3.354(3.354-3.354) | 9.18(7.11-10.562) | Up regulated | 0.935 | 0.865-0.985 |
| hsa-miR-660-5p | 10.176(7.878-11.113) | 12.232(11.963-12.552) | Up regulated | 0.935 | 0.866-0.984 |
| hsa-miR-148b-3p | 11.889(11.476-13.14) | 15.372(14.985-15.721) | Up regulated | 0.931 | 0.815-0.997 |
| hsa-miR-548ad-5p | 7.707(3.354-8.57) | 10.066(9.482-10.603) | Up regulated | 0.928 | 0.861-0.978 |
| hsa-miR-4690-3p | 3.354(3.354-4.422) | 6.637(6.277-7.423) | Up regulated | 0.922 | 0.807-0.997 |
| hsa-miR-499a-5p | 7.393(3.354-8.657) | 12.207(9.393-13.812) | Up regulated | 0.920 | 0.846-0.974 |
| hsa-miR-106b-5p | 13.52(12.952-14.423) | 15.701(14.98-16.152) | Up regulated | 0.917 | 0.845-0.972 |
| hsa-miR-548ae-5p | 6.909(3.354-8.507) | 9.853(9.087-10.349) | Up regulated | 0.914 | 0.838-0.972 |
| hsa-miR-22-5p | 10.603(9.228-11.38) | 12.044(11.657-12.309) | Up regulated | 0.912 | 0.794-0.985 |
| hsa-miR-208a-3p | 3.354(3.354-3.354) | 5.727(4.557-6.965) | Up regulated | 0.905 | 0.836-0.962 |
| hsa-miR-25-3p | 16.222(13.625-17.079) | 17.798(17.177-18.15) | Up regulated | 0.904 | 0.827-0.963 |
| hsa-miR-29c-3p | 10.855(8.993-11.625) | 12.524(12.014-12.749) | Up regulated | 0.902 | 0.821-0.964 |
| hsa-miR-15a-5p | 11.152(9.611-12.397) | 13.84(13.084-14.264) | Up regulated | 0.899 | 0.808-0.966 |
| hsa-miR-548ay-5p | 7.924(3.354-8.924) | 10.06(9.436-10.442) | Up regulated | 0.894 | 0.796-0.968 |
| hsa-miR-101-5p | 6.251(3.354-6.941) | 8.203(7.322-8.68) | Up regulated | 0.892 | 0.805-0.96 |
| hsa-miR-3136-5p | 3.354(3.354-3.354) | 5.84(5.097-6.268) | Up regulated | 0.892 | 0.812-0.955 |
| hsa-miR-3613-5p | 7.053(3.354-8.828) | 10.623(10.139-11.008) | Up regulated | 0.892 | 0.776-0.975 |
| hsa-miR-106a-5p | 9.692(8.602-10.562) | 11.473(11-11.986) | Up regulated | 0.883 | 0.772-0.964 |
| hsa-let-7i-3p | 8.605(5.337-9.5) | 9.953(9.678-10.201) | Up regulated | 0.879 | 0.752-0.974 |
| hsa-miR-196b-3p | 3.354(3.354-3.922) | 5.21(4.821-5.872) | Up regulated | 0.879 | 0.797-0.944 |
| hsa-miR-548ak | 3.354(3.354-5.028) | 6.297(5.451-6.977) | Up regulated | 0.874 | 0.784-0.949 |
| hsa-miR-181a-5p | 14.436(13.647-15.094) | 12.56(12.14-12.806) | Down regulated | 0.873 | 0.702-0.995 |
| hsa-miR-548d-5p | 8.32(3.354-8.976) | 10.053(9.496-10.487) | Up regulated | 0.870 | 0.747-0.962 |
| hsa-miR-6775-3p | 3.354(3.354-3.354) | 5.088(3.813-5.894) | Up regulated | 0.868 | 0.792-0.931 |
| hsa-miR-7855-5p | 3.815(3.354-4.609) | 5.924(5.218-6.366) | Up regulated | 0.868 | 0.753-0.96 |
| hsa-miR-95-5p | 3.354(3.354-4.118) | 5.339(4.697-5.746) | Up regulated | 0.863 | 0.745-0.955 |
| hsa-miR-12136 | 7.478(6.285-10.754) | 5.083(3.691-5.846) | Down regulated | 0.861 | 0.733-0.96 |
| hsa-miR-362-5p | 12.358(11.885-14.025) | 10.337(9.957-10.639) | Down regulated | 0.859 | 0.696-0.987 |
| hsa-miR-1301-3p | 11.562(10.809-12.166) | 9.853(9.613-10.091) | Down regulated | 0.858 | 0.719-0.955 |
| hsa-miR-874-3p | 10.993(9.947-12.37) | 8.844(8.421-9.524) | Down regulated | 0.857 | 0.717-0.956 |
| hsa-miR-99a-5p | 14.919(14.393-16.954) | 13.645(13.237-14.2) | Down regulated | 0.857 | 0.72-0.96 |
| hsa-let-7i-5p | 16.242(15.158-17.125) | 17.493(17.305-17.905) | Up regulated | 0.854 | 0.723-0.952 |
| hsa-miR-4785 | 3.354(3.354-3.825) | 5.52(4.776-5.972) | Up regulated | 0.851 | 0.722-0.95 |

Table S2. Significantly changed miRNAs during ischemia-reperfusion period

| miRNA | VST value [Median(Q1-Q3)] | | | Changes during ischemia- reperfusion period |
| --- | --- | --- | --- | --- |
|  | Control | AMI | Reperfusion |  |
| hsa-miR-101-5p | 6.251(3.354-6.941) | 8.203(7.322-8.68) | 6.933(5.278-7.663) | Up- followed by down-regulated |
| hsa-miR-106a-5p | 9.692(8.602-10.562) | 11.473(11-11.986) | 10.385(9.555-10.941) |  |
| hsa-miR-1-3p | 9.577(7.363-10.769) | 11.937(10.321-13.941) | 9.337(8.421-10.666) |  |
| hsa-miR-143-3p | 14.271(13.559-14.813) | 15.042(14.591-15.67) | 14.724(13.96-15.019) |  |
| hsa-miR-20b-5p | 9.884(8.798-10.986) | 11.788(10.929-12.175) | 10.558(9.409-11.086) |  |
| hsa-miR-369-3p | 4.258(3.354-7.271) | 7.048(5.94-7.976) | 5.034(3.354-6.672) |  |
| hsa-miR-493-5p | 3.815(3.354-6.042) | 6.208(5.332-7.184) | 3.354(3.354-5.559) |  |
| hsa-miR-95-5p | 3.354(3.354-4.118) | 5.339(4.697-5.746) | 6.118(5.012-6.447) | Consecutively up-regulated |
| hsa-miR-199a-3p | 14.498(12.475-14.709) | 13.629(13.308-14.224) | 12.645(11.041-13.432) | Consecutively down-regulated |
| hsa-miR-199a-5p | 12.886(11.879-14.323) | 11.4(11.076-11.978) | 11.109(10.552-11.551) |  |
| hsa-miR-199b-3p | 14.498(12.475-14.709) | 13.629(13.308-14.224) | 12.645(11.041-13.432) |  |
| hsa-miR-2355-5p | 6.993(3.354-8.101) | 6.075(5.496-6.807) | 3.354(3.354-4.18) |  |
| hsa-miR-26a-5p | 16.633(15.554-17.218) | 15.215(14.514-15.746) | 13.451(11.851-15.022) |  |
| hsa-miR-26b-5p | 14.462(12.556-15.424) | 14.505(13.956-15.357) | 12.533(11.341-13.58) |  |
| hsa-miR-30b-5p | 11.706(10.229-13.009) | 12.294(11.87-12.664) | 10.601(8.713-11.669) |  |
| hsa-miR-340-3p | 5.106(3.354-7.423) | 5.574(4.675-6.145) | 3.542(3.354-4.308) |  |
| hsa-miR-374a-5p | 11.255(4.305-13.453) | 10.972(9.858-11.67) | 8.862(8.153-10.315) |  |
| hsa-miR-374b-5p | 8.574(3.354-11.808) | 9.398(8.379-10.106) | 7.35(4.86-9.049) |  |
| hsa-miR-409-3p | 10.498(9.186-10.746) | 8.597(8.239-9.268) | 8.391(7.564-8.874) |  |
| hsa-miR-409-5p | 7.562(3.354-8.52) | 6.03(5.459-6.634) | 4.008(3.354-5.862) |  |
| hsa-miR-495-3p | 7.393(3.354-8.392) | 6.785(6.164-7.127) | 3.776(3.354-5.492) |  |
| hsa-miR-556-5p | 5.321(3.354-6.126) | 3.354(3.354-4.765) | 3.354(3.354-3.354) |  |
| hsa-miR-6515-5p | 6.613(3.354-9.324) | 5.634(5.081-6.108) | 3.672(3.354-5.058) |  |
| hsa-miR-654-3p | 8.576(3.354-9.345) | 7.147(6.686-7.752) | 5.283(3.613-6.621) |  |
| hsa-miR-98-5p | 9.768(8.495-11.079) | 9.443(8.919-10.1) | 8.304(5.902-9.326) |  |
| hsa-miR-1180-3p | 12.522(10.592-12.861) | 10.926(10.547-11.309) | 11.817(11.343-12.645) | Down- followed by up-regulated |
| hsa-miR-1224-5p | 6.947(6.549-11) | 7.589(7.098-8.119) | 8.69(7.827-9.789) |  |
| hsa-miR-122-5p | 18.172(16.487-19.339) | 17.146(16.291-17.839) | 18.051(17.16-19.389) |  |
| hsa-miR-1246 | 11.55(10.265-12.67) | 9.542(9.133-10.038) | 11.791(11.067-12.535) |  |
| hsa-miR-1268a | 4.915(3.66-10.814) | 5.54(4.934-6.149) | 6.701(5.916-8.432) |  |
| hsa-miR-1268b | 4.944(3.66-10.814) | 5.821(5.305-6.316) | 7.17(5.963-8.677) |  |
| hsa-miR-1285-5p | 4.704(3.354-6.104) | 3.354(3.354-4.645) | 5.367(3.354-6.509) |  |
| hsa-miR-1287-5p | 9.167(8.216-11.102) | 8.438(8.016-8.851) | 9.223(8.706-9.739) |  |
| hsa-miR-1290 | 11.08(9.783-12.51) | 8.598(8.277-9.131) | 11.504(10.42-13.088) |  |
| hsa-miR-1291 | 5.901(5.08-6.826) | 3.677(3.354-4.636) | 5.516(3.354-6.636) |  |
| hsa-miR-1292-5p | 8.979(7.883-9.65) | 7.947(7.435-8.377) | 8.235(7.483-8.83) |  |
| hsa-miR-1306-3p | 8.34(7.356-11.426) | 6.548(6.165-6.876) | 7.271(6.539-10.356) |  |
| hsa-miR-1307-5p | 11.975(11.551-13.747) | 11.275(10.973-12.133) | 12.514(11.838-13.493) |  |
| hsa-miR-139-3p | 10.025(8.703-12.421) | 8.128(7.788-8.855) | 8.859(8.386-9.847) |  |
| hsa-miR-148a-5p | 8.915(8.292-11.437) | 8.617(8.102-9.097) | 9.834(8.891-10.369) |  |
| hsa-miR-193a-5p | 12.309(11.742-13.991) | 10.832(10.477-11.571) | 12.918(11.86-13.892) |  |
| hsa-miR-193b-5p | 9.524(9.113-10.898) | 8.844(8.288-9.612) | 11.027(10.122-11.395) |  |
| hsa-miR-1972 | 3.354(3.354-3.98) | 3.354(3.354-3.354) | 3.629(3.354-5.503) |  |
| hsa-miR-210-3p | 11.14(10.584-12.776) | 11.726(11.27-12.029) | 12.352(11.575-13.282) |  |
| hsa-miR-22-3p | 18.881(18.31-19.289) | 18.229(17.994-18.486) | 19.247(18.994-20.716) |  |
| hsa-miR-23a-5p | 9.24(8.33-13.421) | 8.105(7.572-9.004) | 8.638(7.784-11.167) |  |
| hsa-miR-296-3p | 6.097(4.115-10.125) | 5.628(5.047-6.215) | 6.421(5.407-8.322) |  |
| hsa-miR-3168 | 5.007(3.354-9.939) | 3.742(3.354-4.98) | 4.344(3.354-7.737) |  |
| hsa-miR-3177-3p | 5.672(3.354-7.818) | 6.018(5.462-6.409) | 6.333(5.167-6.794) |  |
| hsa-miR-320a-3p | 19.03(18.523-22.874) | 19.083(18.829-19.355) | 19.729(19.307-21.237) |  |
| hsa-miR-320b | 13.7(13.575-17.653) | 13.682(13.382-14.283) | 14.81(14.233-16.301) |  |
| hsa-miR-320c | 12.904(12.43-16.174) | 12.225(11.879-12.837) | 13.759(12.89-15.168) |  |
| hsa-miR-320d | 11.389(10.942-14.487) | 10.603(10.391-11.185) | 12.038(11.526-13.606) |  |
| hsa-miR-320e | 9.879(9.1-12.711) | 9.798(9.381-10.019) | 10.31(9.51-12.031) |  |
| hsa-miR-378a-3p | 14.885(14.558-18.701) | 15.755(15.204-16.196) | 17.179(15.978-18.242) |  |
| hsa-miR-378c | 10.418(9.41-14.651) | 11.868(11.227-12.604) | 14.004(12.383-15.091) |  |
| hsa-miR-378d | 7.907(6.837-11.031) | 9.211(8.476-10.056) | 11.248(9.984-12.921) |  |
| hsa-miR-378f | 6.299(5.669-10.229) | 7.4(6.574-8.08) | 8.718(7.375-9.74) |  |
| hsa-miR-378i | 9.824(9.318-13.543) | 10.079(9.549-10.811) | 11.873(10.706-13.365) |  |
| hsa-miR-3960 | 7.405(6.461-10.415) | 7.504(6.915-8.175) | 9.117(8.228-10.513) |  |
| hsa-miR-422a | 4.622(4.327-7.535) | 5.078(4.678-5.432) | 5.901(4.952-6.552) |  |
| hsa-miR-4429 | 4.527(3.978-6.344) | 4.521(4.303-5.153) | 4.724(4.472-5.678) |  |
| hsa-miR-4508 | 10.666(10.057-14.908) | 11.212(10.472-11.835) | 12.578(11.355-13.936) |  |
| hsa-miR-4726-5p | 4.422(3.354-6.022) | 3.354(3.354-4.626) | 3.833(3.354-6.165) |  |
| hsa-miR-4787-5p | 4.146(3.653-6.347) | 3.354(3.354-4.026) | 4.273(3.354-4.905) |  |
| hsa-miR-483-5p | 12.35(12.139-13.937) | 11.471(11.099-12.174) | 13.937(12.338-14.866) |  |
| hsa-miR-576-3p | 11.135(10.376-14.93) | 10.895(10.54-11.203) | 11.898(11.395-13.015) |  |
| hsa-miR-619-5p | 5.691(4.885-10.043) | 5.34(4.63-5.827) | 6.833(5.761-7.629) |  |
| hsa-miR-627-5p | 7.49(5.971-9.18) | 6.823(6.224-7.291) | 7.284(6.766-8.532) |  |
| hsa-miR-6503-3p | 6.021(5.007-8.59) | 6.034(5.31-6.512) | 6.67(6.148-7.225) |  |
| hsa-miR-671-5p | 8.17(5.669-13.975) | 6.527(5.897-7.292) | 6.783(6.568-9.068) |  |
| hsa-miR-6877-5p | 7.488(6.582-10.014) | 7.381(6.914-7.981) | 7.796(7.2-8.988) |  |
| hsa-miR-873-3p | 7.281(4.924-9.692) | 4.756(3.354-5.436) | 5.38(3.354-6.811) |  |
| hsa-miR-939-5p | 8.062(7.302-11.675) | 7.135(6.777-7.603) | 8.203(6.625-9.858) |  |
| hsa-miR-99a-5p | 14.919(14.393-16.954) | 13.645(13.237-14.2) | 14.403(13.959-15.228) |  |

Table S3. Examination of the independent risk factors for AMI prognosis by multivariate Cox analysis

| Characteristics | Adjusted HR | 95% CI for HR | | *P*-value |
| --- | --- | --- | --- | --- |
|  |  | Lower | Upper |  |
| Reperfusion status |  |  |  |  |
| No | 1 (ref) |  |  |  |
| Yes | 0.1648 | 0.01439 | 1.887 | 0.14718 |
| Survival risk (miR-548ap-5p) |  |  |  |  |
| Low | 1 (ref) |  |  |  |
| High | 11.5101 | 2.03045 | 65.248 | 0.00578 |
| Survival risk (miR-4716-3p) |  |  |  |  |
| Low | 1 (ref) |  |  |  |
| High | 17.3001 | 1.78386 | 167.778 | 0.01392 |
